# Clinical artificial intelligence quality improvement: towards continual monitoring and updating of AI algorithms in healthcare

**DOI:** 10.1038/s41746-022-00611-y

**Published:** 2022-05-31

**Authors:** Jean Feng, Rachael V. Phillips, Ivana Malenica, Andrew Bishara, Alan E. Hubbard, Leo A. Celi, Romain Pirracchio

**Affiliations:** 1 Department of Epidemiology and Biostatistics, University of California, San Francisco, CA USA; 2 Bakar Computational Health Sciences Institute, University of California San Francisco, San Francisco, CA USA; 3 Department of Biostatistics, University of California, Berkeley, CA USA; 4 Department of Anesthesia, University of California, San Francisco, CA USA; 5 Institute for Medical Engineering and Science, Massachusetts Institute of Technology, Department of Medicine, Beth Israel Deaconess Medical Center; Department of Biostatistics, Harvard T.H. Chan School of Public Health, Boston, MA 02115 USA

**Keywords:** Public health, Statistics

## Abstract

Machine learning (ML) and artificial intelligence (AI) algorithms have the potential to derive insights from clinical data and improve patient outcomes. However, these highly complex systems are sensitive to changes in the environment and liable to performance decay. Even after their successful integration into clinical practice, ML/AI algorithms should be continuously monitored and updated to ensure their long-term safety and effectiveness. To bring AI into maturity in clinical care, we advocate for the creation of hospital units responsible for quality assurance and improvement of these algorithms, which we refer to as “AI-QI” units. We discuss how tools that have long been used in hospital quality assurance and quality improvement can be adapted to monitor static ML algorithms. On the other hand, procedures for continual model updating are still nascent. We highlight key considerations when choosing between existing methods and opportunities for methodological innovation.

## Introduction

The use of artificial intelligence (AI) and machine learning (ML) in the clinical arena has developed tremendously over the past decades, with numerous examples in medical imaging, cardiology, and acute care^1–6^. Indeed, the list of AI/ML-based algorithms approved for clinical use by the United States Food and Drug Administration (FDA) continues to grow at a rapid rate^7^. Despite the accelerated development of these medical algorithms, adoption into the clinic has been limited. The challenges encountered on the way to successful integration go far beyond the initial development and evaluation phase. Because ML algorithms are highly data-dependent, a major concern is that their performance depends heavily on how the data are generated in specific contexts, at specific times. It can be difficult to anticipate how these models will behave in real-world settings over time, as their complexity can obscure potential failure modes^8^. Currently, the FDA requires that algorithms not be modified after approval, which we describe as “locked”. Although this policy prevents the introduction of deleterious model updates, locked models are liable to decay in performance over time in highly dynamic environments like healthcare. Indeed, many have documented ML performance decay due to patient case mix, clinical practice patterns, treatment options, and more^9–11^.

To ensure the long-term reliability and effectiveness of AI/ML-based clinical algorithms, it is crucial that we establish systems for regular monitoring and maintenance^12–14^. Although the importance of continual monitoring and updating has been acknowledged in a number of recent papers^15–17^, most articles provide limited details on how to implement such systems. In fact, the most similar work may be recent papers documenting the creation of production-ready ML systems at internet companies^18,19^. Nevertheless, the healthcare setting differs in that errors have more serious repercussions, the number of samples is smaller, and the data tend to be noisier.

**Fig. 1:**
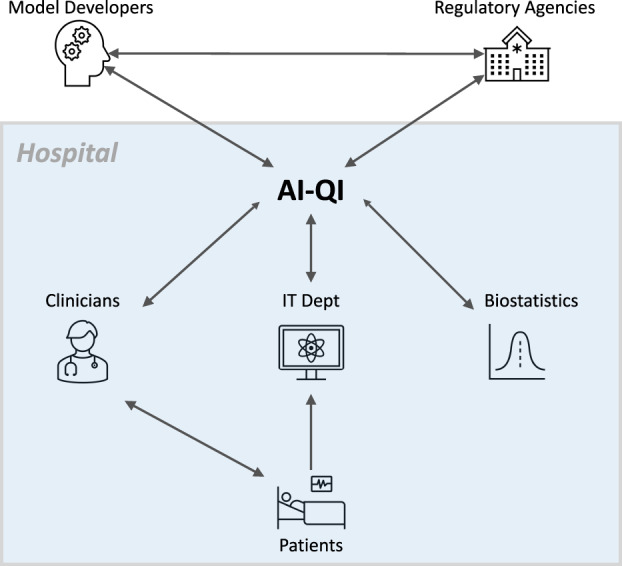
AI-QI is a collaborative effort. To ensure the continued safety and effectiveness of AI-based algorithms deployed in the hospital, institutions will need streamlined processes for monitoring model performance continuously, communicating the latest performance metrics to end-users, and revising the model or even suspending its use when substantial decay in performance is observed. Given its cross-cutting nature, AI-QI requires close collaboration between clinicians, hospital administrators, information technology (IT) professionals, model developers, biostatisticians, and regulatory agencies.

In this work, we look to existing hospital quality assurance (QA) and quality improvement (QI) efforts^20–22^ as a template for designing similar initiatives for clinical AI algorithms, which we refer to as AI-QI. By drawing parallels with standard clinical QI practices, we show how well-established tools from statistical process control (SPC) may be applied to monitoring clinical AI-based algorithms. In addition, we describe a number of unique challenges when monitoring AI algorithms, including a lack of ground truth data, AI-induced treatment-related censoring, and high-dimensionality of the data. Model updating is a new task altogether, with many opportunities for technical innovations. We outline key considerations and tradeoffs when selecting between model updating procedures. Effective implementation of AI-QI will require close collaboration between clinicians, hospital administrators, information technology (IT) professionals, biostatisticians, model developers, and regulatory agencies (Fig. 1). Finally, to ground our discussion, we will use the example of a hypothetical AI-based early warning system for acute hypotensive episodes (AHEs), inspired by the FDA-approved Edwards’ Acumen Hypotension Prediction Index^23^.

## Error in clinical AI algorithms

As defined by the Center for Medicare and Medicaid Services, Quality Improvement (QI) is the framework used to systematically improve care through the use of standardized processes and structures to reduce variation, achieve predictable results, and improve outcomes for patients, healthcare systems, and organizations. In this section we describe why clinical AI algorithms can fail and why a structured and integrated AI-QI process is necessary.

Simply put, AI-based algorithms achieve high predictive accuracy by detecting correlations between patient variables and outcomes. For example, a model that forecasts imminent AHE may rely on patterns in physiological signals that commonly occur prior to such an event, such as a general downward trend in blood pressure and a rise in heart rate. Correlation-based models tend to have good internal validity: they work well when the target population is similar to the training data. However, when the clinical environment is highly dynamic and patient populations are heterogeneous, a model that works well in one-time period or one hospital may fail in another. A recent example is the emergence of COVID-19^24^ documented a performance drop in an ML algorithm for determining which patients were at high risk of hospital admission based on their emergency department (ED) presentation that relied on input variables like respiratory rate and arrival mode, which were significantly affected by the spread of COVID-19.

### Different causes of error

Per the QI literature, variability in system-level performance is due to either “common-cause” or “special-cause” variation. *Common-cause variation* refers to predictable and unavoidable variability in the system. Continuing with our AHE example, an algorithm that predicts future mean arterial pressure (MAP) levels is bound to make errors because of inherent variability in the physiological parameter; this error is acceptable as long as it matches specifications from the manufacturer, e.g. the observed and predicted MAP are expected to be within 5 mmHg 95% of the time. Prior to model deployment, developers can calibrate the model and characterize common-cause variation using independent data^25–27^. Model developers can also incorporate known sources of common-cause variation into the model to improve its generalizability^28,29^.

On the other hand, *special-cause variation* represents unexpected change in the system. In our AHE example, this may occur if the hospital follows new guidelines for managing hypotension, leading to a change in the association between future MAP levels and medication history. Using statistical terminology, special-cause variations are unexpected drops in performance due to shifts in the joint distribution of the model inputs *X* and the target variable(s) *Y*, which are more succinctly referred to as *distribution* or *dataset shifts*^30^. In general, distribution shifts can be categorized based on which relationships have changed in the data, such as changes solely in the distribution of the input variables *X* versus changes in the conditional distribution of *Y* given *X*.

Different types of distribution shifts need to be handled differently. Sometimes, impending distribution shifts can be anticipated, such as well-communicated hospital-wide policy changes. To stay informed of these types of changes, AI-QI efforts can take a proactive approach by staying abreast of hospital’s current events and subscribing to mailing lists. Hospital administrators and clinicians can help interpret the impact that these changes will have on the ML algorithm’s performance. Other distribution shifts are unannounced and can be more subtle. To detect these changes as quickly as possible, one will need procedures for monitoring the ML algorithm’s performance.

Special-cause variation can also be characterized as *sustained* or *isolated* (i.e. those that affect a single observation). The focus in this manuscript is on the former, which can degrade performance for significant periods of time. The detection of such system-level shifts typically cannot be accomplished by analyzing each observation individually and instead require analyzing a stream of observations. In contrast, isolated errors can be viewed as outliers and can be targeted using Shewhart control charts^31^, a popular technique in SPC, as well as general outlier detection methods^32^.

### Cause-and-effect diagrams

**Fig. 2:**
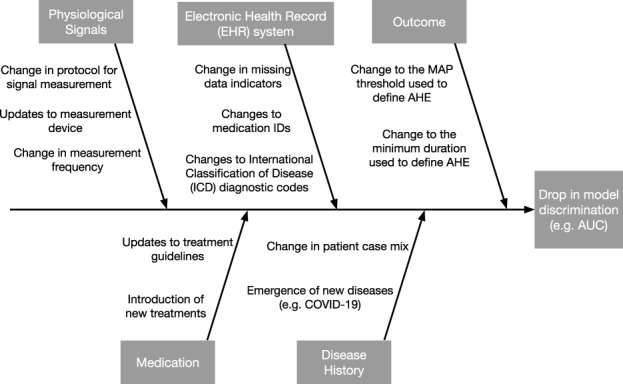
Cause-and-effect diagram for a drop in performance of an AI-based early warning system for Acute Hypotension Episodes (AHEs). Each branch represents a category of potential causes. The effect is defined as model performance, which is measured by the area under the receiver operating characteristic curve (AUC).

When the reasons for a drop in system performance are unclear, the cause-and-effect diagram—also known as the fishbone or Ishikawa diagram—is a formal tool in QI that can help unlayer the potential causes^31^. The “head" of the diagram is the effect, which is a drop in model performance. Potential causes are listed on the branches, grouped by the major categories. We show an example cause-and-effect diagram for an AHE early warning system in Fig. 2. Cause-and-effect diagrams in QI share many similarities to causal Directed Acyclic Graphs from the causal inference literature^33^. Indeed, a recent idea developed independently by the ML community is to use causal diagrams to understand how different types of dataset shifts can impact model performance^29,34^.

Generally speaking, we can categorize potential causes of a performance drop into (i) changes in the distribution of the target variable *Y*, (ii) changes in the distribution of model inputs *X*, and (iii) changes in the relationship between *X* and *Y*. Using statistical terminology, (i) and (ii) refer to shifts in the *marginal distribution* of *Y* and *X*, respectively, and (iii) refers to shifts in the *conditional distribution* of *Y*∣*X* or *X*∣*Y*. These potential causes can further divided based on semantically meaningful subgroups of the model inputs, such as physiological signals measured using the same device. While one should describe changes pertaining to every input variable, particular attention should be paid to those assigned high feature importance, as shifts in such features are more likely to induce larger shifts in performance.

## Monitoring clinical AI algorithms

The goal in AI monitoring is to raise an alarm when special-cause variation is present and help teams identify necessary corrections to the model or the data generation/collection process. Both common-cause and special-cause variation can cause drops in performance, so statistical procedures are needed to distinguish between the two. Here we introduce *statistical control charts*, a standard tool in SPC to help visualize and detect different types of shifts. This section focuses on locked models; we will discuss evolving algorithms later.

Given a stream of observations, a typical control chart plots a summary statistic over time and displays control limits to indicate the normal range of values for this statistic. When the chart statistic exceeds the control limits, an alarm is fired to indicate the likely existence of special-cause variation. After an alarm has been fired, the hospital should investigate the root cause and determine whether corrective actions need to be taken and if so, which ones. This requires a close collaboration of many entities, including the original model developer, healthcare providers, IT professionals, and statisticians.

Carefully designed control charts ensure the rate of false alarms is below some prespecified threshold while minimizing the delay in detecting important changes. Statistical support is needed to help make decisions on which procedures are most appropriate and how to implement them.

Next, we describe methods for detecting shifts in the marginal distribution of *Y*; this is the simplest mathematically speaking, because *Y* is typically low-dimensional. Building on this, we describe methods for detecting shifts in the marginal distribution of *X*, followed by those for conditional distributions. Table 1 presents a summary of the methods described in this section.

**Table 1:** Methods from statistical process control (SPC) and their application to monitoring ML algorithms.

Method(s)	What the method(s) detect and assumptions	Example uses
CUSUM, EWMA	Detects a shift in the mean of a single variable, given shift size. Assumes the pre-shift mean and variance are known. Extensions can monitor changes in the variance.	• Monitoring changes in individual input variables
		• Monitoring changes in real-valued performance metrics (e.g. monitoring the prediction error)
MCUSUM, MEWMA, Hotelling’s T^2^	Monitor changes in the relationship between multiple variables	• Monitoring changes in the relationship between input variables
Generalized likelihood ratio test (GLRT), Online change point detection	Detects if a change occurred in a data distribution and when. Can be applied if characteristics of the pre- and/or post-shift distributions are unknown. GLRT methods typically make parametric assumptions. Parametric and nonparametric variants exist for online change point detection methods.	• Detecting distributional shifts for individual or multiple input variables
		• Detecting shifts in the conditional distribution of outcome *Y* given input variables
		• Determining whether parametric model recalibration/revision is needed
Generalized fluctuation monitoring	Monitor changes to the residuals or gradient	• Detect when the average gradient of the training loss for a differentiable ML algorithm (e.g. neural network) differs from zero

### Monitoring changes in the target variable

When labeled data are available, one can use control charts to monitor changes in the distribution of *Y*. For a one-dimensional outcome *Y*, we can use univariate control charts to monitor changes in summary statistics such as the mean, variance, and rate of missingness. In the context of our AHE example, we can use this to monitor changes in the prevalence of AHE or the average MAP value. If *Y* is a vector of multiple outcomes, a simple solution is to construct separate control charts for each one. Commonly used control charts that fall in this category include Shewhart control charts, cumulative sum (CUSUM) control charts^35^, and exponentially weighted moving average (EWMA) control charts^31^. In practice, the distribution of *Y* may be subject to many sources of variation such as seasonality. One solution is to model the expected value of each observation given known sources of variability and apply SPC methods to monitor the residuals.

### Monitoring changes in the input variables

Statistical control charts can also be used to monitor changes in the marginal distribution of the input variables. A major advantage of these charts is that they can be readily implemented even when the outcome is difficult to measure or can only be observed after a long delay.

We have already described univariate control charts in the previous section; these can also be used to monitor the input variables individually. When it is important to monitor the relationship between the input variables, one should instead use multivariate control charts such as the multivariate CUSUM and EWMA (MCUSUM and MEWMA, respectively) and Hotelling’s T^2^ ^36^. If *X* is high dimensional, traditional SPC methods can have inflated false alarm rates or low power to detect changes. This can be addressed using variable selection^37^, dimension reduction techniques^38^, or histogram binning^39^. For complex data types like physiological waveforms, medical images, and clinical notes, representation learning methods can transform the data into a lower-dimensional vector that is suitable for inputting into traditional control charts^40,41^. Fundamental to detecting distribution shifts is the quantification of distance between two distributions. Recent work has proposed new distance measures between high-dimensional multivariate probability distributions, such as the Wasserstein distance, f-divergences^42^, and kernel-based measures^43,44^.

Given the complexity of ML algorithms, a number of papers have suggested monitoring ML explainability metrics, such as variable importance (VI)^18,24^. The idea is that these metrics provide a more interpretable representation of the data. Nevertheless, it is important not to over-interpret these charts. Because most VI metrics defined in the ML literature quantify the importance of each feature as attributed by the existing model, shifts in these metrics simply indicate a change in the distribution of the input variables; they do not necessarily indicate if and how the relationship between the input and target variables has changed. For example, an increase in the average VI of a given variable indicates that its distribution has shifted towards values that are *assigned* higher importance, but that variable may have actually become less predictive of *Y*. To monitor population-level variable importance instead^45^, we suggest monitoring the relationship between *X* and *Y* using techniques described in the following section.

### Monitoring changes in the relationship between the input and target variables

Finally, statistical control charts can be used to monitor changes in the relationship between *X* and *Y*. The most intuitive approach, perhaps, is to monitor performance metrics that were used to train or test the original model^46^. In the AHE example, one may choose to monitor the mean squared error (MSE) between the predicted and observed MAP values or the area under the receiver operating characteristic curve (AUC) given predicted AHE risks and the observed AHE events. By tracking a variety of such metrics, different aspects of prediction performance can be measured, such as model discrimination, calibration, and fairness. Performance metrics that are defined as the average loss over individual observations (e.g. MSE) can be monitored using univariate control charts as described in the previous section. Performance metrics that can only be estimated using a batch of observations (e.g. AUC) require grouping together observations and monitoring batch-wise summaries instead.

While procedures for monitoring performance metrics are simple and intuitive, their major drawback is that the performance can drop due to changes in either the marginal or the conditional distributions. For example, a drop in the prediction accuracy of our AHE early warning system can be due to either a change in the patient population (a shift in *X*) or a change in the epidemiology (a shift in *Y*∣*X*). To guide the root cause analysis, it is important to distinguish between the two. Next, we describe procedures for detecting if a change has occurred solely in the conditional distributions.

To monitor changes in the conditional distribution *Y*∣*X*, one can apply generalizations of the CUSUM procedure such as the Shiryaev-Roberts procedure^47,48^ and the generalized likelihood ratio test (GLRT)^49,50^. Briefly, these methods monitor differences between the original model and the refitted model for a candidate change point. By monitoring the difference between these two models, these methods are only sensitive to changes in the conditional distribution. Furthermore, one can consider a broader class of so-called generalized M-fluctuation tests that gives the user more flexibility in deciding which metrics to track^51^. When deciding between monitoring procedures, it is important to understand the underlying assumptions. For instance, procedures for monitoring parametric models cannot be used to directly monitor complex AI algorithms such as neural networks, but can be used to monitor parametric *recalibration* models (e.g. logistic recalibration^52^). Recent works have looked to relax common assumptions, including nonparametric extensions^53,54^ and methods for handling high-dimensional *X*^55–57^.

In certain cases, one may instead be interested in monitoring *X*∣*Y*. This is relevant, for instance, when the ML algorithm predicts disease diagnosis *Y* given a radiographic image *X*, because the disease may manifest differently over time and the resulting images may change. If *Y* takes on only a few values, one can individually monitor the distribution of *X* within each strata using methods described in the previous section. If *Y* takes on many values or is continuous, one can use the aforementioned procedures for monitoring changes in *Y*∣*X*, where we switch the ordering of *X* and *Y*. For high-dimensional *X*, one should apply dimension reduction prior to the application of these methods and monitor the conditional relationship between the reduced features and *Y* instead.

### Challenges of monitoring clinical AI algorithms

Despite the growing utilization of control charts in healthcare, it is important to recognize that many of these methods were originally developed for industrial manufacturing, where the data is much more uniform and one has much finer control over the data collection process. Prior work has described how to address differences between health-related control chart applications and industrial applications^58^. New challenges and opportunities arise when these methods are used to monitor clinical AI algorithms. Here we present two such challenges, but there are many more that we will be unable to touch upon in this manuscript.

One major challenge faced in many settings is the latency between the predictions being generated by the algorithm and the target variable. For example, outcomes such as mortality or the development of a secondary malignancy typically require a significant follow-up period. In such cases, it becomes difficult to respond to changes in algorithm performance in a timely fashion. A potential solution is to monitor how well an AI algorithm predicts surrogate outcomes. Changes in this proxy measure would serve as a “canary” that something has gone wrong. As an example, consider an algorithm designed to predict 30-day patient survival. We can monitor the algorithm’s AUC for predicting a closer endpoint such as 5-day patient survival to shorten the detection delay. Model developers can also facilitate AI-QI by providing algorithms that output predictions for both the outcome of interest and these surrogate outcomes. We note that surrogate outcomes in the context of AI-QI do not necessarily need to satisfy the same formal properties used to measure treatment efficacy^59,60^, because the cost of a false alarm is much lower in our setting.

Another challenge is AI-induced confounding. That is, when AI-based algorithms provide clinically actionable predictions, clinicians may choose to adjust their treatment plan based on the algorithm’s predictions. Returning to our example of an AHE early warning system, if the ML algorithm generates an alert that an AHE is likely to occur within the next 30 min, the hospital staff may decide to administer treatment via fluids and/or vasopressors in response. If the patient doesn’t experience a hypotensive episode 30 min later, a question emerges: was the algorithm wrong, or did the prescribed intervention change the circumstances? In such situations, we must account for the role of human factors^61^ and confounding medical interventions (CMIs), because we cannot observe the counterfactual outcome that would have occurred if the prediction were not available. Although confounding occurs in the absence of AI-based predictions^62,63^, the CMIs becomes much more severe when clinicians utilize AI algorithms in their decision-making process^64–66^. In fact, the more effective the AI is, the faster the AI algorithm’s performance will appear to degrade.

From the statistical perspective, the best approach to obtaining an unbiased estimate of the model’s performance is to randomly select a subset of patients for whom providers do not receive AI-based predictions. However, the ethics of such an approach need to be examined and only minor variations on standard of care are typically considered in hospital QI. Another option is to rely on missing data and causal inference techniques to adjust for confounding^66,67^. While this sidesteps the issue of medical ethics, causal inference methods depend on strong assumptions to make valid conclusions. This can be tenuous when analyzing data streams, since such methods require the assumptions to hold at all time points. There are currently no definitive solutions and more research is warranted.

### Example: Monitoring an early warning system for acute hypotension episodes

**Fig. 3:**
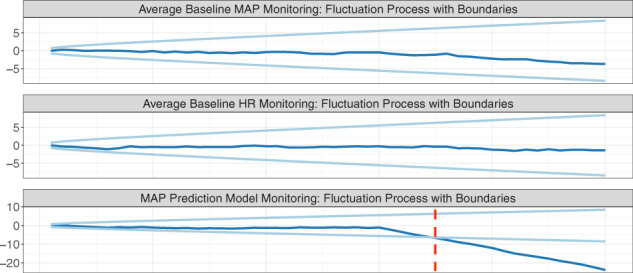
Continual monitoring of a hypothetical AI algorithm for forecasting mean arterial pressure (MAP). Consider a hypothetical MAP prediction algorithm that predicts a patient’s risk of developing an acute hypotensive episode based on two input variables: baseline MAP and heart rate (HR). The top two rows monitors changes in the two input variables using the CUSUM procedure, where the dark line is the chart statistic and the light lines are the control limits. The third row aims to detect changes in the conditional relationship between the outcome and input variables by monitoring the residuals using the CUSUM procedure. An alarm is fired when a chart statistic exceeds its control limits.

Here we present a simulation to illustrate how SPC can be used to monitor the performance of an AHE early warning system (Fig. 3). Suppose the algorithm forecasts future MAP levels and relies on baseline MAP and heart rate (HR) as input variables. The clinician is notified when MAP is predicted to fall below 65 mmHg in the next 15 min.

In the simulation, we observe a new patient at each time point. Two shifts occur at time point 30: we introduce a small shift to the average baseline MAP, and a larger shift in the conditional relationship between the outcome and the two input variables. We construct control charts to detect changes in the mean baseline MAP and HR and the conditional relationship *Y*∣*X*. Using the monitoring software provided by the strucchange R package^68^, we construct control limits such that the false alarm rate is 0.05 in each of the control charts. The chart statistic crosses the control limits at time 35, corresponding to a delay of five time points. After an alarm is fired, the hospital should initiate a root cause analysis. Referring to the cause-and-effect diagram in Fig. 2, one may conclude that the conditional relationship has changed due to a change in epidemiology, such as the emergence of COVID-19 in the patient population. If this change in the conditional relationship is expected to be persistent, the AI-QI team will likely need to update the model.

## Updating clinical AI/ML algorithms

The aim of model updating is to correct for observed drops in model performance, prevent such drops from occurring, and even improve model performance over time. By analyzing a stream of patient data and outcomes, these procedures have the potential to continuously adapt to distribution shifts. We note that in contrast to AI monitoring, model updating procedures do not necessarily have to discriminate between common- versus special-cause variation. Nevertheless, it is often helpful to understand which type of variation is being targeted by each modification, since this can elucidate whether further corrective actions need to be taken (e.g. updating data pre-processing rather than the model).

Model updating procedures cannot be taken lightly, since there is always a risk that the proposed modifications degrade performance instead. Given the complexities of continual model updating, current real-world updates to clinical prediction model have generally been confined to ad-hoc one-time updates^69,70^. Still, the long-term usability of AI algorithms relies on having procedures that introduce regular model updates that are guaranteed to be safe and effective. In light of this, regulatory agencies are now considering various solutions for this so-called “update problem”^71^. For instance, the US FDA has proposed that the model vendor provide an Algorithm Change Protocol (ACP), a document that describes how modifications will be generated and validated^15^. This framework is aligned with the European Medicines Agency’s policies for general medical devices, which already require vendors to provide change management plans and perform post-market surveillance^72^.

Below we highlight some of the key considerations when designing/selecting a model updating procedure. Table 2 presents a summary of the methods described below.

**Table 2:** Model updating procedures described in this paper. The performance guarantees from these methods require the stream of data to be IID with respect to the target population. Note that in general, online learning methods may provide only weak performance guarantees or none at all.

Method(s)	Update frequency	Complexity of model update	Performance guarantees
One-time model recalibration (e.g. Platt scaling, isotonic regression, temperature scaling)	Low	Low	Strong
One-time model revision	Low	Medium	Strong
One-time model refitting	Low	High	Strong
Online hypothesis testing for approving proposed modifications	Medium	High	Strong
Online parametric model recalibration/revision	High	Low/Medium	Medium

### Performance metrics

The choice of performance metrics is crucial in model updating, just as they are in ML monitoring. The reason is that model updating procedures that provide guarantees with respect to one set of performance metrics may not protect against degradation of others. For example, many results in the online learning literature provide guarantees that the performance of the evolving model will be better than the original model on average across the target population, over some multi-year time period. Although this provides a first level of defense against ML performance decay, such guarantees do not mean that the the evolving model will be superior within every subpopulation nor at every time point. As such, it is important to understand how performance is quantified by the online learning procedure and what guarantees it provides. Statistical support will be necessary to ensure the selected model updating procedure meets desired performance requirements.

Another example arises in the setting of predictive policing, in which an algorithm tries to allocate police across a city to prevent crimes:^73^ showed how continual retraining of the algorithm on observed crime data, along with a naïve performance metric, can lead to runaway feedback loops where police are repeatedly sent back to the same neighborhoods regardless of the true crime rate. These challenges have spurred research to design performance metrics that maintain or even promote algorithmic fairness and are resistant to the creation of deleterious feedback loops^74–76^.

### Complexity of model updates

When deciding between different types of model updates, one must consider their “model complexities” and the bias-variance tradeoff^77,78^. The simplest type of model update is recalibration, in which continuous scores (e.g. predicted risks) produced by the original model are mapped to new values; examples include Platt scaling, temperature scaling, and isotonic regression^79–82^. More extensive model revisions transform predictions from the original model by taking into account other variables. For example, logistic model revision regresses the outcome against the prediction from the original model and other shift-prone variables^83^. This category also includes procedures that fine-tune only the top layer of a neural network.

The most complex model updates are those that retrain the model from scratch or fit an entirely different model. There is a tradeoff when opting for higher complexity: one is better able to protect against complex distribution shifts, but the resulting updates are sensitive to noise in the data and, without careful control of model complexity, can be overfit. Because data velocities in medical settings tend to be slow, simple model updates can often be highly effective^84^.

Nevertheless, more complex model updates may eventually be useful as more data continues to accumulate. Procedures like online cross-validation^85^ and Bayesian model averaging^86^ can help one dynamically select the most appropriate model complexity over time.

### Frequency of model updates

Another design consideration is deciding when and how often model updates occur. Broadly speaking, two approaches exist: a “reactive” approach, which updates the model only in response to issues detected by continual monitoring versus a “continual updating” approach, which updates the model even if no issues have been detected. The latter is much less common in clinical practice, though there have been multiple calls for regular model updating^87–89^. The advantage of continual updating is that they can improve (not just maintain) model performance, respond quickly to changes in the environment, reduce the number of patients exposed to a badly performing algorithm, and potentially improve clinician trust.

Nevertheless, there are many challenges in implementing continual updating procedures^13^. For instance, procedures that retrain models on only the most recent data can exhibit a phenomenon known as “catastrophic forgetting”, in which the integration of new data into the model can overwrite knowledge learned in the past. On the other hand, procedures that retrain models on all previously collected data can fail to adapt to important temporal shifts and are computationally expensive. To decide how much data should be used to retrain the model, one can simulate the online learning procedure on retrospective data to assess the risk of catastrophic forgetting and the relevance of past data (see e.g.^10^). Another challenge is that many online updating methods fail to provide meaningful performance guarantees over realistic time horizons. Theoretical guarantees for updating complex ML algorithms like neural networks are particularly difficult to establish. Instead, recent work has proposed to employ “meta-procedures” that approve modifications proposed by a black-box online learning procedure and ensure the approved modifications satisfy certain performance guarantees. Among such methods, online hypothesis testing provides strongest guarantees^90,91^. Another approach is to use continual updating procedures for parametric models, for whom theoretical properties *can* be derived, for the purposes of model revision, such as in online logistic recalibration/revision^92^ and online model averaging^93^.

### Quality of model update data

The performance of learned model updates depends on the quality of the training data. As such, many published studies of one-time model updates have relied on hand-curating training data and performing extensive data validation^69,87^. This process can be highly labor-intensive. For instance,^70^ described how careful experimental design was necessary to update a risk prediction model for delirium among patients in the intensive care unit. Because the outcome was subjective, one needed to consider typical issues of inter- and intra-rater reliability. In addition, predictions from the deployed AI algorithm could bias outcome assessment, so the assessors had to be blinded to the algorithm and its predictions.

Nonetheless, as model updates increase in frequency, there will be a need for more automated data collection and cleaning. Unfortunately, the most readily available data streams in medical settings are observational in nature and subject to confounding, structural biases, missingness, and misclassification of outcomes, among others^94,95^. More research is needed to understand how models can continually learn from real-world data streams. Support from clinicians and the IT department will be crucial to understanding data provenance and how it may impact online learning procedures.

## Discussion

To bring clinical AI into maturity, AI systems must be continually monitored and updated. We described general statistical frameworks for monitoring algorithmic performance and key considerations when designing model updating procedures. In discussing AI-QI, we have highlighted how it is a cross-cutting initiative that requires collaboration between model developers, clinicians, IT professionals, biostatisticians, and regulatory agencies. To spearhead this effort, we urge clinical enterprises to create AI-QI teams who will spearhead the continual monitoring and maintenance of AI/ML systems. By serving as the “glue” between these different entities, AI-QI teams will improve the safety and effectiveness of these algorithms not only at the hospital level but also at the national or multi-national level.

Clinical QI initiatives are usually led at the department/division level. Because AI-QI requires many types of expertise and resources outside those available to any specific clinical department, we believe that AI-QI entities should span clinical departments. Such a group can be hosted by existing structures, such as a department of Biostatistics or Epidemiology. Alternatively, hospitals may look to create dedicated Clinical AI departments, which would centralize efforts to develop, deploy, and maintain AI models in clinical care^96^. Regardless of where this unit is hosted, the success of this team will depend on having key analytical capabilities, such as structured data acquisition, data governance, statistical and machine learning expertise, and clinical workflow integration. Much of this assumes the hospital has reached a sufficient level of analytical maturity (see e.g. HIMSS “Adoption Model for Analytics Maturity”) and builds upon tools developed by the hospital IT department. Indeed, the IT department will be a key partner in building these data pipelines and surfacing model performance measures in the clinician workstation.

When deciding whether to adopt an AI system into clinical practice, it will also be important for hospitals to clarify how the responsibilities of model monitoring and updating will be divided between the model developer and the AI-QI team. This is particularly relevant when the algorithm is proprietary; the division of responsibility can be more flexible when the algorithm is developed by an internal team. For example, how should the model be designed to facilitate monitoring and what tools should a model vendor provide for monitoring their algorithm? Likewise, what tools and training data should the model vendor provide for updating the model? One option is that the model vendor takes full responsibility for providing these tools to the AI-QI team. The advantage of this option is that it minimizes the burden on the AI-QI team and the model vendor can leverage data from multiple institutions to improve model monitoring and maintenance^97,98^. Nevertheless, this raises potential issues of conflicts of interest, as the model vendor is now responsible for monitoring the performance of their own product. A second option is for the local AI-QI unit at the hospital to take complete responsibility. The advantage of this is that the hospital has full freedom over the monitoring pipeline, such as choosing the metrics that are most relevant. The disadvantage, however, is that one can no longer leverage data from other institutions, which can be particularly useful for learning good algorithmic modifications. A third and most likely option is that the responsibility is shared between the hospital’s AI-QI team and the model vendor. For example, the hospitals take on the responsibility of introducing site-specific adjustments, and the manufacturer takes on the responsibility of deploying more extensive model updates that can only be learned using data across multiple sites.

In addition to hospital-level monitoring by the AI-QI team, regulatory agencies will be instrumental in ensuring the long-term safety and effectiveness of AI-based algorithms at the national or international level. Current proposals require algorithm vendors to spearhead performance monitoring^15^. Although the vendor will certainly play a major role in designing the monitoring pipeline, the monitoring procedure itself should be conducted by an independent entity to avoid conflicts of interest. To this end, existing post-market surveillance systems like the FDA’s Sentinel Initiative^99^ could be adapted to monitor AI-based algorithms in healthcare, extending the scope of these programs to not only include pharmacosurveillance but “technovigilence”^100,101^. Moreover, AI-QI teams can serve as key partners in this nationwide initiative, by sharing data and insights on local model performance. If substantial drift in performance is detected across multiple sites, the regulatory agency should have the ability to put the AI algorithm’s license on hold.

In general, there are very few studies that have evaluated the effectiveness of continuous monitoring and maintenance methods for AI-based algorithms applied to medical data streams, perhaps due to a dearth of public datasets with timestamps. Most studies have considered either simulated data or data from a single, private medical dataset^52,92,93^. Although large publicly available datasets such as the Medical Information Mart for Intensive Care (MIMIC) database^102^ are moving in the direction of releasing more accurate timestamps, random date shifts used for data de-identification have the unfortunate side effect of dampening temporal shifts extant in the data. How one can validate ML monitoring and updating procedures on time-stamped data while preserving patient privacy remains an open problem.

Finally, there are currently few software packages available for the monitoring and maintenance of AI algorithms^103–105^. Those that do exist are limited, either in the types of algorithms, data types, and/or the statistical guarantees they offer. There is a pressing need to create robust open-source software packages for AI-QI and facilitate hospitals along their journey to become *AI ready*.

## Supplementary information

Supplementary Information: Code for simulation

## Data Availability

Data sharing not applicable to this article as no datasets were generated or analyzed during the current study.

## Code availability

Code for the example of monitoring an AHE early warning system is included in the Supplementary Materials.
